# Cancer-Associated Fibroblasts: Tumorigenicity and Targeting for Cancer Therapy

**DOI:** 10.3390/cancers14163906

**Published:** 2022-08-12

**Authors:** Raisa A. Glabman, Peter L. Choyke, Noriko Sato

**Affiliations:** 1 Molecular Imaging Branch, Center for Cancer Research, National Cancer Institute, National Institutes of Health, Bethesda, MD 20892, USA; 2 Department of Comparative Medicine and Integrative Biology, College of Veterinary Medicine, Michigan State University, East Lansing, MI 48824, USA

**Keywords:** cancer-associated fibroblasts, cancer therapy, cancer-associated fibroblast targeting, tumor microenvironment

## Abstract

**Simple Summary:**

Cancer-associated fibroblasts (CAFs) are found in the tumor microenvironment and exhibit several protumorigenic functions. Preclinical studies suggest that CAFs can be reduced, eliminated, or reprogrammed; however, clinical translation has not yet occurred. A better understanding of these cells and their functions will undoubtedly improve cancer treatments. In this review, we summarize current research, highlight major challenges, and discuss future opportunities for improving our knowledge of CAF biology and targeting.

**Abstract:**

Cancer-associated fibroblasts (CAFs) are a heterogenous group of activated fibroblasts and a major component of the tumor stroma. CAFs may be derived from fibroblasts, epithelial cells, endothelial cells, cancer stem cells, adipocytes, pericytes, or stellate cells. These complex origins may underlie their functional diversity, which includes pro-tumorigenic roles in extracellular matrix remodeling, the suppression of anti-tumor immunity, and resistance to cancer therapy. Several methods for targeting CAFs to inhibit tumor progression and enhance anti-tumor immunity have recently been reported. While preclinical studies have shown promise, to date they have been unsuccessful in human clinical trials against melanoma, breast cancer, pancreas cancer, and colorectal cancers. This review summarizes recent and major advances in CAF-targeting therapies, including DNA-based vaccines, anti-CAF CAR-T cells, and modifying and reprogramming CAF functions. The challenges in developing effective anti-CAF treatment are highlighted, which include CAF heterogeneity and plasticity, the lack of specific target markers for CAFs, the limitations in animal models recapitulating the human cancer microenvironment, and the undesirable off-target and systemic side effects. Overcoming these challenges and expanding our understanding of the basic biology of CAFs is necessary for making progress towards safe and effective therapeutic strategies against cancers in human patients.

## 1. Introduction

Over 1.9 million new cancer cases are expected in the United States in 2022, and solid tumors comprise approximately 90% of these cases [1]. As we continue to expand our knowledge of cancer, we now recognize the tumor microenvironment as a heterogenous, intricate system composed of tumor cells and nonmalignant host components including immune cells, stroma, and vasculature, which shapes the nature of the tumor. In many epithelial tumors, including pancreatic, lung, breast, and colorectal cancers, the stroma can comprise up to 90% of the cancer mass [2]. Within the tumor stroma are both cellular and noncellular components, including collagen, fibroblasts, and mesenchymal stromal cells that provide structure and remodel the tissue. Activated stromal tissue in the pathological context forms desmoplasia or fibrosis, resulting in increased mass and stiffness, which are considered negative prognostic indicators. Fibroblasts constitute one of the most abundant and critical cell types in the tumor stroma and are the major producers of connective tissue extracellular matrix (ECM). Within the tumor microenvironment, various inflammatory cytokines produced by cancer cells, host immune and stromal cells induce the activation of fibroblasts. These activated fibroblasts are termed cancer-associated fibroblasts (CAFs). Through their production of soluble factors, such as cytokines and chemokines, and ECM, CAFs strongly influence surrounding cells. They support tumor progression and metastasis by promoting cancer cell growth, enhancing pro-tumor immune responses, remodeling the ECM, influencing tumor cell drug resistance, and promoting angiogenesis [3,4]. In this review, we focus on CAFs, discuss their biologic tumor-promoting functions and recent advancements in the development of CAF-targeting cancer therapies.

## 2. Definitions, Origins and Basic Biology of CAFs

### 2.1. Definition of CAFs

Fibroblasts are found in virtually all organs and normal tissues and contribute to inflammation and fibrosis during wound healing. CAFs are activated fibroblasts with a mesenchymal lineage associated with cancer and contribute to tumor-promoting inflammation and fibrosis. CAFs are defined by a combination of their morphology, association with cancer cells, and lack of lineage markers for epithelial cells, endothelial cells, and hematopoietic cells [4]. CAFs maintain key roles in regulating the biologic function of the tumor stroma and contribute to immune regulation, angiogenesis, and ECM remodeling of the tumor, as well as the generation and maintenance of cancer stem cells, thereby promoting therapeutic resistance.

### 2.2. Distinction of CAFs from Resting Fibroblasts

CAFs differ in several respects from resting fibroblasts residing in normal tissues. CAFs are generally larger than resting fibroblasts, spindle-shaped, and have indented nuclei and branching cytoplasm. However, the difference between the two is largely a functional distinction. CAFs possess enhanced proliferative, migratory, and secretory properties. CAFs are more metabolically active than untransformed fibroblasts, producing increased extracellular matrix factors such as tenascin, periostin (POSTN), and secreted protein acidic and rich in cysteine (SPARC) [5]. Collagen production by CAFs is abnormal, characterized by increased production and often a more rigid and contractile pattern of collagen deposition [6,7,8]. Several signaling mechanisms are recognized in the transition from resting fibroblast to CAF, including the activation of the Hippo pathway, the loss of p53, and the activation of heat shock factor protein 1 triggered by inflammation and changes in the structure and composition of the ECM [6,9]. CAFs are even found in circulation, akin to circulating tumor cells (CTCs)-circulating CAFs (identified based on the expression of fibroblast activation protein (FAP) and alpha-smooth muscle actin (α-SMA)), and were found in 88% of patients with metastatic breast cancer and in 23% of patients with nonmetastatic disease [9], suggesting a role in metastasis and formation of the pre-metastatic niche.

### 2.3. Origin of CAFs

CAFs can arise from a myriad of cell precursors, which can also vary between tissues. The origins of all CAFs are not entirely and fully elucidated. Regardless of origin, the transition to CAF is largely irreversible, and yet remains plastic with regard to the CAF phenotype within or across tumor types. CAFs often develop from local resident fibroblast populations but can also differentiate from mesenchymal stromal cells or mesenchymal stem cells (MSCs). MSCs express a similar, less abundant set of surface markers, and possess the ability to differentiate into osteoblasts, chondrocytes, and adipocytes [10,11,12]. Quiescent resident fibroblasts in the liver and pancreas, also known as pancreatic stellate cells, can acquire a CAF phenotype upon activation by tumor growth factor beta (TGFβ) and platelet-derived growth factors (PDGFs) [13,14]. Outside of the fibroblast lineage, CAFs can transdifferentiate from epithelial cells, blood vessels, adipocytes, pericytes, and smooth muscle cells via endothelial to mesenchymal transition (EMT) and endothelial to mesenchymal transition (EndMT). Fibrocytes, circulating mesenchymal cells derived from monocyte precursors, can also become CAFs [15]. Both noninvasive and invasive cancer cells can express the EMT markers β-catenin and vimentin or S100A4, so these are also not unique to CAFs. Importantly, CAFs can secrete proinflammatory cytokines, such as interleukin (IL)-6, which promotes the EMT of cancer cells, forming a positive feedback loop [16,17,18]. The recruitment and activation of CAFs is mediated by hypoxic conditions, oxidative stress, and certain growth factors produced by tumor cells. TGF-β, epidermal growth factor (EGF), fibroblast growth factor type 2 (FGF2), and PDGF are known to act as key regulators of CAF recruitment and activation [19,20]. Additionally, IL-1β from innate immune cells triggers NF-kB activation and production of IL-6 in CAFs via the JAK-STAT pathway, contributing to CAF differentiation [21]. Lysophosphatidic acid produced by cancer cells synergizes with TGF-β to drive the activation and increase the contractility of CAFs [4]. Recent research has also shown that exosomes secreted by murine melanoma, human squamous cell carcinoma, and human breast carcinoma can promote the differentiation of fibroblasts into CAFs, mediated by TGF-β and downstream SMAD signaling pathways [22,23]. Overall, the precise origin and roles of fibroblast populations within the tumor microenvironment remain poorly understood. Further studies using lineage tracing for the cell of origin [24,25] will be essential in deepening our understanding on the origins of CAFs, as well as their evolution during tumorigenesis.

### 2.4. CAF Phenotypic and Functional Heterogeneity

Tumors are spatially and functionally heterogeneous ecosystems [26], and the variety of sources from which CAFs can arise lend complexity to their phenotype, gene expression, and function. Several biomarkers have been established to detect CAFs, however none are completely exclusive. To date, CAFs are defined as cells that lack the expression of biomarkers for epithelial, endothelial, or hematopoietic cells but express mesenchymal biomarkers such as vimentin, α-SMA, FAP, and platelet-derived growth factor receptor alpha (PDGFR-α), and lack genetic mutations [27,28]. As the phenotypes of CAFs differ between tumor type, CAF studies necessitate the combined application of multiple biomarkers for the detection and identification of these cells. As a result, CAFs are often identified by a combination of α-SMA, tenascin-C, periostin (POSTN), NG2 chondroitin sulfate proteoglycan, PDGFRα/β, and FAP expression. Other mesenchymal markers include vimentin, fibronectin, type I collagen, prolyl 4-hydroxylase, fibroblast surface protein, and fibroblast specific protein-1 (FSP-1)/S100A4 [29]. Biomarkers expressed by both normal fibroblasts and CAFs are summarized in Table 1.

Historically, activated fibroblasts expressing α-SMA were termed ‘myofibroblasts’, but are now recognized to be only one subset among several within the tumor microenvironment. **α-SMA+ CAFs** predominantly produce and modulate the ECM, facilitate cell–ECM adhesion, and regulate adaptive immunity [59]. **α-SMA+ CAFs** are also located more distally to tumor cells. **FAP+ CAFs** are immunosuppressive with increased ECM alignment and stiffness, and this is hypothesized to be a major factor in the transition from a tumor-resistant to tumor-permissive microenvironment [75]. Stiffness of the tumor stroma influences invasion through tumor cell integrin-dependent mechanotransduction signaling [76], and is correlated with increased metastasis [77,78].

Newer analytic methods such as single-cell RNA sequencing (scRNAseq) and cytometry by time of flight (cyTOF) have begun to help answer questions concerning functional subsets. Functional CAF subsets maintain unique cytokine expression profiles that variably shape the tumor microenvironment. While some CAF subsets do not seem to affect immune cell populations, others in fact, modulate the immune microenvironment, often in pro-tumorigenic ways. The most recent scRNAseq transcriptome data suggest there are between 3 and 7 major subsets of fibroblasts [52,79,80], but some of these groups may have overlapping features, as well as context-dependent and tumor-dependent variability. Nonetheless, there is growing evidence for similar or shared phenotypes across tumor types as discussed in the following sections.

### 2.5. Functional CAF Subsets in Human Cancers

The analysis of distinct CAF subpopulations at the single cell level has largely been performed in the context of human pancreatic cancer, with several studies also examining these cells in other human tumor types (Table 2). To highlight some of these classifications, in human pancreatic cancer, at least two major CAF phenotypes are defined by their expression of α-SMA and IL-6. A more matrix-secreting, TGF-β–responsive, high-α-SMA, and low-cytokine (e.g., IL-6, IL-11)-expressing myofibroblastic, **myCAF** population, and inflammatory-type, **iCAFs**, that exhibit high IL-6 and IL-11 production and low α-SMA expression [51,52,60,81]. Spatial distribution of these two populations also differs—myCAFs are often found adjacent to neoplastic cells whereas iCAFs localize within dense stromal regions distant from neoplastic cells [60]. Interestingly, pancreatic CAFs, formerly quiescent pancreatic stellate cells, are able to transition between the **myCAF** and **iCAF** states, although the mechanism by which this occurs is not well understood [60]. A third CAF phenotype, **apCAFs,** are characterized by major histocompatibility complex (MHC) class II and CD74 expression and are capable of presenting antigens to CD4 T cells, but lack classical costimulatory molecules expressed by professional antigen-presenting cells [52]. **MyCAF** and **iCAF** subpopulations have also been identified in human cholangiocarcinoma [82] and bladder cancer [83]. Human triple-negative breast [45,84] and ovarian [85] cancer studies have yielded 3–4 CAF subtypes, designated **CAF S1–S4** based on the differential expression of six fibroblast markers (FAP, integrin β1/CD29, α-SMA, S100-A4/FSP1, PDGFRβ, and caveolin-1). Other phenotyping studies in human lung [80], prostate [86], head and neck [87], and colorectal [88,89] cancers similarly classify CAF subpopulations based on high versus low α-SMA expression and/or functional characteristics.

### 2.6. Functional CAF Subsets in Murine Cancers

The three CAF subsets described in human tumors are also found in murine pancreatic cancer models by scRNAseq analysis; ECM-producing **myCAFs**, inflammatory **iCAFs**, and a third smaller population of antigen-presenting **apCAFs.** CAF subsets in spontaneous mouse mammary tumor models (the MMTV-PyVT mouse model) have been categorized into four main groups, vascular CAFs (**vCAFs**), cycling CAFs (**cCAF**), matrix CAFs (**mCAF**), and developmental CAFs (**dCAF**) [93]. **vCAFs** are angiogenic, predominantly located near vessels and thought to arise from perivascular cell precursors. **cCAFs** are considered the proliferating fraction of vCAFs, with similar transcriptional profiles as vCAFs, exhibiting upregulated cell cycle genes (e.g., *Nuf2*, *Mki67*, *Ccna2*, *Top2a*, *and Cep55*). **mCAFs** are descendants of resident fibroblasts, expressing fibulin-1 and PDGFRα, which are often positioned at the invasive front of tumors. **dCAFs** express development-associated genes and are similar in phenotype and are proximal to cancer cells, suggesting that they may originate from a malignant cell precursor. In 4T1 mouse mammary tumor models, eight subtypes of CAFs divided into in two main populations, **pCAF** and sCAF, are described based on the selective expression of PDPN or S100A4. The ratio of pCAFs and sCAFs changes with tumor progression and is associated with disease outcome in triple-negative breast cancer patients [95].

CAFs have been functionally categorized in other murine cancers, such as melanoma, as either immune (**S1**), desmoplastic (**S2**), or contractile (**S3**) [90], and in cholangiocarcinoma as either **myCAF**, **iCAF**, or mesothelial **mesCAF** [82]. Overall, the existence of both myofibroblastic and inflammatory CAF populations appears to be the most consistent observation in both human and mouse tumor models. Across cancer types, **myCAFs** are associated with high ECM production, whereas non-myofibroblastic **iCAFs** are generally characterized by a secretory, inflammatory phenotype. Lastly, CAFs can also be grouped based on location, e.g., primary tumor, circulation, or metastasis [96,97].

### 2.7. Challenges in Defining and Detecting CAFs

By far, one of the greatest challenges in defining CAFs is the lack of a pan-specific biomarker. In addition, no standardization nor consensus of biomarkers to identify CAFs currently exist, adding to the difficulty in differentiating CAFs from other mesenchymal cells (e.g., adipocytes or pericytes). This lack of uniform analysis makes interpretation of previous studies and understanding of the full biological implications of these cells difficult. Standardized functional and molecular definitions of fibroblast subtypes also do not yet exist. There is inherent plasticity between CAF subtypes, suggesting these are functional fibroblastic states, as opposed to static fibroblast types, adding to their complexity [98]. CAFs continue to evolve over time and eventually differentiate into subpopulations that promote tumor development in ways that are not only tissue specific but tumor specific. Identifying what triggers this plasticity will also be invaluable in future research, as phenotypic or functional subsets may not function comparably across tumor types. It is increasingly clear that the tumor microenvironment changes throughout cancer progression, and likely so do CAFs. Longitudinal studies, particularly focused on CAF plasticity, are necessary for further insight.

## 3. Protumorigenic Functions of CAFs

Various components of the tumor microenvironment promote tumor progression and resistance to cancer therapy. For instance, mesenchymal stem cells can secrete vascular endothelial growth factor (VEGF), promoting vessel growth, and prostaglandin E2 (PGE2), impeding the anti-tumor immune response through the suppression of T cell function. Pericytes and adipocytes can produce pro-tumorigenic growth factors and cytokines and even contribute to T cell anergy [99]. Finally, immune cells such as tumor-associated macrophages (TAMs) can promote EMT and inflammation-associated angiogenesis [100]. Here, we focus specifically on the roles of CAFs.

### 3.1. Tumor-Promoting Secretory Factors

**Figure 1:**
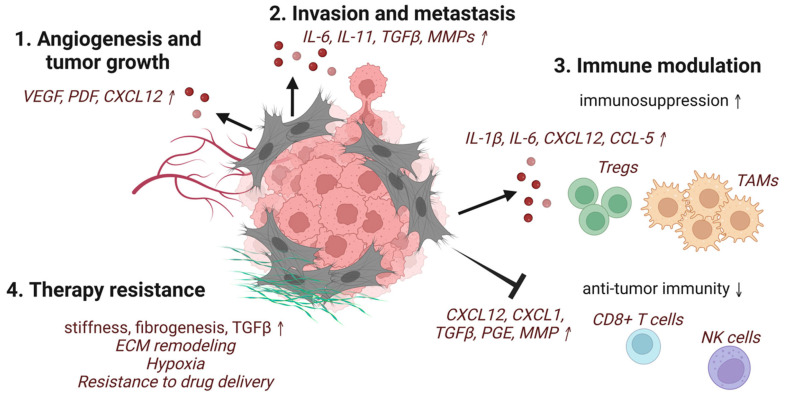
Schematic representation of selected pro-tumorigenic functions of CAFs. CAFs induce (**1**) angiogenesis and tumor growth, (**2**) invasion and metastasis of cancer cells, (**3**) modulation of the immune system, including recruitment and activation of immune suppressors and inhibition of anti-tumor effector cells, and (**4**) therapy-resistance through ECM production and remodeling (created with BioRender.com accessed 19 June 2022).

In general, CAFs secrete far more cytokines and chemokines than their resting counterparts. These secreted factors include TGFβ, PDGF, FGF, hepatocyte growth factor (HGF), VEGF, tumor necrosis factor α (TNFα), interferon-γ (IFNγ), CXCL12, IL-6, connective tissue growth factor (CTGFβ), EGF, growth arrest-specific protein 6 (GAS6), galectin-1, secreted frizzled-related protein 1 (SFRP1), sonic hedgehog protein (SHH), and bone morphogenetic protein (BMP), which are tumor-promoting (Figure 1) [6]. As the master regulator of fibrosis and a major secreted factor of CAFs, TGFβ predominantly mediates crosstalk between CAFs and cancer cells. The inhibition of TGFβ signaling using a number of approaches has been shown to significantly inhibit tumor growth and metastasis [101].

CAFs have also been demonstrated to induce EMT and promote the growth and migration of cancer cells via IL-6 [102,103]. Elevated levels of CAF-derived IL-6 induces the activation of the JAK/STAT3 signaling pathway, leading to tumor cell proliferation mediated by the activation of cyclin D1, among other cell cycle mediators. Tumor survival is enhanced by the activation of downstream BCL2-like protein 1 (BCL-xL). STAT3 also induces the expression of angiogenic factor VEGF. During tumor neovascularization, degradation of the basement membrane and ECM occurs, with a contribution from matrix metalloproteinases (MMPs) [104], to allow for endothelial cells to migrate and generate new vessels. This process, in turn, enhances cancer invasion and metastasis. The hyperactivation of STAT3 in anti-tumor immune cells exerts a negative regulatory effect that also contributes to an immunosuppressive tumor microenvironment [105]. Other signaling pathways governing the tumor-promoting ability of CAFs include PDGF-PDGFR, which acts through paracrine signaling on cancer cells to drive tumor growth [29].

### 3.2. Resistance to Chemotherapies and Radiation

Classic chemotherapy targets rapidly proliferating cells, but does not eliminate all CAFs, nor those cancer cells that become drug resistant. CAFs can also contribute to the development of resistant cancer phenotypes following cycles of chemotherapies. Several in vitro experiments demonstrate that DNA damage induced by chemotherapies prompted increased cancer cell invasion and survival through stromal-derived paracrine signaling via cytokines and exosomes [6]. For example, this occurs via glial-derived neurotrophic factor (GDNF) production in prostate cancer [106], IL-6 in lymphoma [107], and exosome secretion in colorectal cancer [108]. Chemotherapy-induced genotoxic stress can also trigger a DNA damage secretory program resulting in the release of numerous inflammatory (IL-6/8), angiogenic (VEGF, CXCL1), mitogenic (amphiregulin), and pro-EMT (HGF) factors [98].

Several chemotherapy drugs have been reported to induce CAF-like phenotypes in resting fibroblasts and promote stemness in breast [109] and colorectal cancers [108]. This is thought to occur following an exposure of cancer cells to a hypoxic environment, which activates hypoxia-inducible factor (HIF-1α) and sonic hedgehog-GLI signaling [109,110]. CAF-mediated TGF-β signaling synergizes with HIF-1α signaling and enhances the expression of GLI2 in cancer cells, inducing stemness. This results in a resistance to chemotherapy. In fact, a high expression of the HIF-1α/TGF-β is associated with an increased risk of colorectal cancer recurrence in patients undergoing chemotherapy [110]. Similarly, studies have found that CAFs contribute to drug resistance and reduce the efficacy of anti-EGFR cetuximab [111], gemcitabine [112], and the tyrosine kinase inhibitor gefitinib [113].

As with the examples of chemotherapies described above, radiation therapy impedes cancer cell growth through DNA damage. Radiation affects not only cancer cells but also the tissue microenvironment surrounding cancer cells, which includes immune cells, endothelial cells, vasculature, and fibroblasts. Fibroblasts are highly resistant to radiation, even at high doses. Irradiated fibroblasts can overcome apoptotic signals and become senescent but have also been demonstrated to convert to a more activated CAF phenotype [114,115,116]. In one study, radiation exposure activated CAFs and upregulated their expression of CXCL12, which directly acted on pancreatic cancer cells via CXCR4, promoting EMT and invasion in vitro and in vivo [117]. The enhanced expression of CXCL12, HGF, MMPs, and TGF-β in irradiated fibroblasts was found to increase invasion and EMT in cancer cells as indicated by the increased expression of vimentin, snail and beta-catenin, and decreased E-cadherin expression. [114,115,116].

The CAF-directed resistance to radiotherapy and post-radiation recurrence of cancers is reported to be associated with activation of the autophagy pathway. It is likely this response is at least in part related to CAF-secreted IGF1/2, CXCL12, and β-hydroxybutyrate, leading to increased reactive oxygen species (ROS), enhancing protein phosphatase 2A (PP2A) activity, repressing mTOR activation, and ultimately resulting in autophagy in cancer cells after irradiation [118,119]. The IGF2 neutralizing antibody and autophagy inhibitor 3-MA consistently reduced the CAF-promoted tumor relapse in tumor-bearing mice after radiotherapy [118]. Combining CAF-targeted therapies and chemotherapy or radiation could yield a more powerful and robust anti-tumor response.

### 3.3. Immunomodulatory Role of CAFs

Advances in immune checkpoint inhibitors, such as PD-1 and CTLA-4, have brought much attention to the immune cell–tumor crosstalk; however, less is known about the contribution of stromal components to the immune milieu. Recent studies suggest CAFs mediate the tumor immune landscape via the secretion of various cytokines, growth factors, chemokines, exosomes, and other effector molecules, ultimately shaping an immunosuppressive tumor microenvironment, enabling cancer cells to evade immune surveillance, and limiting immunotherapy strategies.

In general, CAFs shape the tumor microenvironment by the production of proinflammatory cytokines, including IL-1β and IL-6 [21,120], and express the ligands CXCL12 [30], CXCL1 [121], and G-CSF [122] that can drive downstream immunosuppressive signaling pathways. For instance, CXCL12 regulates interactions between tumor cells and surrounding cells in the tumor microenvironment, promoting tumor survival, proliferation, angiogenesis, and metastasis. It also promotes the recruitment of immunosuppressive cells and their precursors, notably bone marrow mesenchymal stem cells and monocytes that differentiate into TAMs. Inflammatory CAFs, or iCAFs, highly express CXCL12, which binds to CXCR4 [52]. Blocking the CXCL12-CXCR4 interaction can induce cancer regression in pre-clinical models [30,123]. CAFs also interact with T cells, NK cells, dendritic cells (DCs), myeloid-derived suppressor cells (MDSCs), mast cells, tumor-associated neutrophils (TANs) and TAMs in the tumor microenvironment, generally resulting in an immunopermissive environment.

CAFs prevent CD8+ cytotoxic T cell activity and recruitment within tumors, in part through TGF-β [124,125,126] and CXCL12 [127]. Both TGF-β c and CXCL12 are known to contribute to cytotoxic T cell exclusion by attenuation of the anti–PD-L1 response [30]. While limiting anti-tumor cytotoxic T cells, CAFs can also increase intratumoral Treg recruitment and scRNAseq revealed the upregulation of PD-1 and CTLA4 in Tregs. CAFs appear to attract, help accumulate, and promote the survival of FOXP3+ Tregs in human triple-negative breast cancer [84]. FoxP3+ Tregs are known to restrain host-antitumor-immunity, and thereby lend an unfavorable prognosis in a number of cancers. Although the precise mechanism of crosstalk between Tregs and CAFs remains unclear, high numbers of both cell types are found in stromal regions and are associated with low survival in cancers such as lung adenocarcinoma [128].

NK cells are well-known innate effector cells; however, their function can be impaired by CAFs through the inhibition of NK receptor activation, cytotoxic activity, and cytokine production [129,130]. Netrin G1 (NetG1) expression on CAFs can suppress the cytotoxic function of NK cells and support the survival of cancer cells in nutrient-deprived environments and is thus linked to a poor prognosis in cancers such as pancreatic ductal adenocarcinoma [131]. Tumor-infiltrating DCs are also critical to the anti-tumor immune response, and their functionality can similarly be impaired by CAFs. By activating the IL-6-mediated STAT3 pathway, CAFs in hepatocellular carcinoma transdifferentiated DCs into regulatory DCs (rDCs) that produced inhibitory cytokines and enzymes such as indoleamine 2,3-dioxygenase (IDO) [132]. VEGF produced by CAFs is also involved in the abnormal differentiation and impaired antigen presenting function of DCs via inhibition of NF-κB activation [133].

MDSCs can be also be recruited to the tumor microenvironment by CAFs via CCL2 [134], thereby suppressing CD8 T cell proliferation and IFN-γ production [135]. Mast cells, which can be both tumor-suppressing and tumor-promoting, can be recruited by CAFs via CXCL12 in a CXCR4-dependent manner [136]. In vitro, mast cells and CAFs can act together to induce the malignant transformation of benign epithelial cells [137]. Furthermore, N2, or protumorigenic polarization of neutrophils within the tumor can be induced through CAF-derived cardiotrophin-like cytokine factor 1 (CLCF1), which upregulates CXCL6 and TGF-β on tumor cells [138]. Neutrophils may also be directly recruited by CAFs through the secretion of CXCL12 or the expression of CXCR2, thus becoming TANs [139,140]. CAFs regulate the activation, survival, and function of TANs through the IL-6/STAT3-PDL1 signaling axis [139].

Like other cells in the tumor microenvironment, TAMs and CAFs have synergistic effects and are often detected in similar areas of tumor tissue. Their combined presence is a negative prognostic predictive indicator in human cancers [141]. Likewise, CAFs are involved in monocyte recruitment, macrophage differentiation, and polarization toward tumor-promoting, or an M2 phenotype [142,143], through the secretion of macrophage colony-stimulating factor 1 (M-CSF1), IL-6, CCL2 [144], and IL-8 [145]. M2 macrophages are reciprocally able to stimulate CAF activation through IL-6 and CXCL12 [142]. While research accumulates on the interactions of CAFs and immune cells in the tumor microenvironment, many ongoing questions remain unanswered. Undoubtedly, understanding CAF and immune cell interactions will provide the basis for novel strategies for targeted immunotherapies.

## 4. Targeting CAFs: Anti-Cancer Therapies

Significant advances have been made in CAF-targeted therapies in recent years. Predominantly, these methods aim to (1) directly or indirectly deplete CAFs, (2) reduce or eliminate the tumor-promoting and immunosuppressive functions of CAFs, or (3) normalize or reprogram CAFs to a more quiescent state. Those strategies are summarized here.

### 4.1. Chemotherapy Targeting CAFs

As discussed previously, FAP is expressed on subsets of CAFs in various tumors. FAP is a membrane-bound serine postprolyl peptidase that differs from other dipeptidyl prolyl peptidases in that it also has endopeptidase activity [146]. A competitive inhibitor of prolyl peptidase, Val-boroPro (Talabostat) is an oral drug that showed some tumor growth control by degrading ECM in mice [147]. However, in human clinical trials for metastatic colorectal cancers, no therapeutic efficacy was observed [148]. Sibrotuzumab is a humanized anti-FAP monoclonal antibody (clone F19) that inhibits the dipeptidyl peptidase activity of FAP [149]. Unlike F19, Sibrotuzumab did not demonstrate inhibitory activity and failed to suppress the growth of pancreatic cancers in patients, despite documented evidence of accumulation of the antibody in the tumor using a radiolabeled version of the antibody (iodine-131-labeled Sibrotuzumab) imaged by single photon emission computed tomography (SPECT) [150].

Taking advantage of the unique enzymatic activity of FAP, anti-CAF prodrugs or protoxins contain cytotoxic agents coupled with a dipeptide containing a FAP cleavage site [146,151]. These prodrugs remain inactive when systemically delivered and are proteolytically activated upon cleavage by FAP, which is expressed on CAFs in the tumor. Intratumoral injection of these prodrugs produced tumor lysis and growth inhibition in human breast and prostate cancer xenografts [146,151,152]. Another class of drugs are the immunotoxins that use an antibody to specifically deliver a toxin to the target cells. Anti-FAP-PE39 immunotoxin suppressed mammary tumor growth and increased the recruitment of tumor-infiltrating lymphocytes [153]. A monoclonal antibody conjugated with a tubulin binding drug maytrasinoid and a bispecific antibody simultaneously targeting FAP on CAFs and death receptor 5 on tumor cells has shown potent anti-tumor effects [153,154]. In another strategy, nanoparticles such as FAP-targeted liposomes have been explored as carriers to specifically deliver therapeutic drugs (e.g., doxorubicin, anti-Tenascin C) to CAFs [155,156] or to remodel the tumor microenvironment [157,158]. Despite the success of preclinical strategies, including substantially attenuating the growth of tumor xenografts in various cancer models with minimal to no toxicity [152,159,160,161], clinical translation is still in its early stages.

### 4.2. Immunotherapy

Various strategies to enhance immunity against FAP-expressing cells (i.e., CAFs) and to suppress cancer growth have been explored. Vaccination against FAP using dendritic cells transfected with FAP mRNA led to the suppressed growth of implanted and intravenously injected tumors [162]. The efficacy was enhanced when a co-vaccination against FAP and a tumor cell-associated antigen was used. These DC vaccines, synergistically combined with an anti-fibrotic agent, showed promising activation of both innate and adaptive immunity. Enhanced NK cell activity, anti-tumoral humoral immunity, and a cytotoxic CD8+ T cell response was observed in three different tumor models [162]. Similarly, adenoviral anti-FAP vaccines are able to selectively deplete CAFs by stimulating a CD8+ T cell response, leading to the inhibition of tumor growth and metastasis in several murine cancer models [163,164,165,166]. In a landmark study using a transgenic mouse expressing the diphtheria toxin receptor under the FAP promoter, depleting FAP+ CAFs by diphtheria toxin administration improved anti-cancer vaccination efficacy [167]. An orally administered anti-FAP DNA vaccine notably suppressed neoangiogenesis, tumor growth, and the metastasis of orthotopically injected breast carcinoma cells [163]. Adding doxorubicin substantially increased the intratumoral uptake of the drug and prolonged lifespans of vaccinated mice [168].

Adoptive chimeric antigen receptor (CAR)-T cell therapy can also be used to directly target CAFs [159,166,169]. FAP-specific CAR-T cells deplete most FAP+ cells, including CAFs, and restrict tumor stroma generation, resulting in the improved uptake and anti-tumor effects of chemotherapeutic drugs. Unfortunately, several studies have observed severe side effects using this approach, such as significant bone marrow toxicity and cachexia [170,171]. More selective and yet unknown targets may improve the precision of CAF-based therapies, which remains an active field of research [50].

Finally, near-infrared photoimmunotherapy (NIR-PIT) is an innovative approach to CAF depletion that has been used to directly and specifically deplete FAP-expressing cells in the tumor microenvironment. Tumor growth was inhibited using a co-culture xenograft model of human esophageal squamous cell carcinoma without adverse effects [172]. Anti-FAP+ CAF therapy combined with 5-fluorouracil (5-FU) could overcome chemoresistance compared with 5-FU alone [173].

### 4.3. Functional Modification/Reprogramming

Strategies that aim to revert activated CAFs to quiescence include the use of all-trans-retinoic acid (ATRA) [174,175,176], minnelide (which de-regulates the TGF-β signaling pathway) [177,178], and calcipotriol, a vitamin D receptor ligand [179,180]. The angiotensin receptor II antagonist losartan has been shown to decrease TGF-β-mediated activation of CAFs, reducing desmoplasia and increasing drug delivery and efficacy of immunotherapy [181,182,183]. Losartan, in combination with other traditional chemotherapies to treat pancreatic cancer, is currently under investigation in clinical trials [184]. Recent strategies seek to block immunosuppressive ligands of major CAF signaling pathways such as IL-6 [185,186], LIF [187], and TGF-β [124,126] in order to suppress or kill cancer cells.

The CXCL12/CXCR4 axis is important in cancer progression and immunosuppression. CXCL12 produced by CAFs recruits CXCR4-expressing endothelial progenitor cells and immune suppressive Tregs, which contributes to angiogenesis and tumor growth [45,127]. The abrogation of CXCR4 signaling in CAFs using the CXCR4 inhibitor plerixafor significantly reduced fibrosis, leading to vasculature normalization, increased cytotoxic T cell infiltration, decreased immunosuppressive cell populations, and increased checkpoint inhibitor efficacy [123]. Other strategies that inhibit CAF functions include TGF-β blockade [188], NFkB inhibitors to overcome chemotherapy resistance [189], and Smoothened (SMO) hedgehog pathway inhibitors (IPI-926) [190].

## 5. Future Perspectives

CAFs play an integral role in the promotion of tumor growth. However, the origin and functional roles of unique CAF subsets are yet to be fully understood, as well as their niche within various tumor types. Determining the spatial and temporal dynamics of CAFs and their cell-to-cell interactions in the tumor microenvironment will add critical information to our knowledge on these fascinating cells. While much of CAF biology has been modeled in vitro, it has been repeatedly demonstrated that CAFs in culture do not fully recapitulate the heterogeneity of CAFs in vivo [87,191,192]. Increasing the strategic use of animal models, including humanized and genetically engineered mouse models, is critical to further understanding the origin, plasticity, and phenotypes of CAFs over time. To this end, the future of the field will undoubtedly include new and emerging technologies such as fate mapping and scRNAseq to assess changes in both stromal cells and immune cells through tumor progression, digital or multiplex spatial profiling of proteins or RNA in tissue to assess spatial changes in the tumor microenvironment, spatial transcriptomics, digital pathology and three-dimensional tissue clearing, and 3D culture systems. Intravital microscopy will provide live visualization of cell-to-cell interactions in vivo. These technologies will bring breakthroughs, such as the identification of even more CAF subpopulations, their cellular interactions, and further insights into CAF heterogeneity and plasticity.

In addition to these analytic methods, a consensus on CAF biomarkers will need to be reached, so that similar phenotypes may be compared across tumor types and preclinical models used in different laboratories. Improving therapeutic delivery methods, such as targeted CAF therapy, rather than stromal-directed therapy, is now becoming more common. FAP shows promise as a CAF marker for CAR-targeted therapy. Recently emerged FAP imaging using gallium-68 labeled small-molecule FAP inhibitor (FAPI) as a tracer for positron emission tomography (PET) suggests superiority in detecting FAP+ cell-containing cancers in patients compared with [fluorine-18]fluoro-deoxy-glucose PET in various cancers [193]. FAPI PET could be used to predetermine the candidacy for anti-FAP CAF-targeted therapies as well as to evaluate the therapy efficacy.

## 6. Conclusions

This review summarizes key advances in CAF-directed therapies and highlights new techniques for the molecular targeting of CAFs. Although a dominant cell type in the tumor microenvironment, CAFs are difficult to precisely target for therapy because of their heterogeneity. These challenges must be overcome to have a meaningful impact from benchtop to clinical intervention. The origins of CAFs across cancer types remains elusive, as does the complete picture of subtypes and functional heterogeneity. Minimizing off target and systemic effects is an ongoing challenge. Finally, the combination of CAF immunotherapies with existing therapies may be valuable and remains an active area of investigation. These methods potentially add further insights to our knowledge of CAF biology but can also help improve precision cancer therapeutics and patient outcomes.

## Figures and Tables

**Table 1:** Biomarkers used to identify fibroblasts and cancer-associated fibroblasts.

Marker	Localization	Expressed by	Role in Tumor Functionality/ Progression	References
Fibroblast Activation Protein (FAP)	Membrane	Fibroblasts, immune cells	Tumor progression and metastasis, shaping the immunosuppressive TME, ECM remodeling, fibrogenesis	[30,31,32,33]
Platelet derived growth factor receptor α/β (PDGFRα/β)	Membrane	Fibroblasts, vascular smooth muscle cells, pericytes	M2 polarization, angiogenesis	[27,34,35]
Podoplanin (PDPN)	Membrane	Endothelial cells	Immunosuppression, tumor growth	[36,37,38,39,40]
α11β1 integrin (ITGA11)	Membrane	Mesenchymal cells	Cancer cell migration, adhesion, tumor cell invasion, desmoplasia	[41,42,43,44]
Caveolin-1 (CAV1)	Membrane	Endothelial cells, epithelial cells, adipocytes, smooth muscle cells, pneumocytes	Vascular and pleural invasion of cancer cells, metastasis	[45,46,47,48,49]
CD10	Membrane	Bone marrow mesenchymal stem cells, pre-B lymphocytes	Sustaining cancer stemness, cancer formation, chemoresistance	[50]
CD74	Membrane	Fibroblasts, monocytes, macrophages, epithelial cells	Antigen presentation	[51,52]
Ly6C	Membrane	Inflammatory CAFs, myeloid cells	Protumorigenic inflammation	[51,52]
Thy-1 (CD90)	Membrane	Fibroblasts, neurons, endothelial cells, tumor cells, immune cells	Tumor cell invasion, migration, tumor-associated endothelial cells	[53,54,55,56]
Vimentin	Cytoplasmic	Fibroblasts, mesenchymal cells	Tumor growth, invasion, migration, endothelial to mesenchymal transition	[57,58]
α-smooth muscle actin (α-SMA)	Cytoplasmic	Fibroblasts, smooth muscle cells	Tumor cell proliferation, protection mechanism, impediment to drug delivery, ECM remodeling, desmoplasia, cancer stemness	[45,59,60]
FSP-1/S100A4	Cytoplasmic, nuclear	Fibroblasts, epithelial and endothelial cells	Promotion of metastasis, immune evasion, immune surveillance, cell motility, fibrosis	[35,61,62,63]
Tenascin-C	ECM protein	Fibroblasts, tumor cells, endothelial cells	Driver of metastasis, Epithelial–mesenchymal transition, desmoplasia, angiogenesis	[64,65,66]
Periostin (POSTN, OSF-2)	ECM protein	Fibroblasts, tumor cells, mesenchymal stem cells	Cancer cell stemness, promotes tumor progression and metastasis	[67,68,69,70]
Type-I collagen (COL1) and COL11α1	Cytoplasmic	Fibroblasts, tumor cells, endothelial cells (COL1), COL11α1 considered to be highly CAF-specific	Epithelial–mesenchymal transition, metastasis	[35,71,72,73,74]

TME: tumor microenvironment, ECM: extracellular matrix.

**Table 2:** Cancer-associated fibroblast subtypes across different cancers.

Tumor Type	Species	CAF Subtype	Relevant Biomarker (s) or Major Feature (s)	Reference (s):
Pancreatic cancer	Patient samples, Murine tumors (KPC)	myCAF–ECM producing	FAP, α-SMA^hi^, Thy1, TAGLN	[51,52,60,79]
		iCAF-inflammatory	Ly6C^hi,^ α-SMA^lo^, PDGFRα^hi^, IL-1, IL-6	
		ApCAF–Ag presenting	MHCII	
Colorectal cancer	Patient samples	CAF-A	α-SMA^lo^, FAP, MMP2, DCN, ECM remodeling	[88,89]
		CAF-B	α-SMA^hi^, TAGLN^hi^, PDGFRα, FAP-; activated myofibroblasts	
Head and neck cancer	Patient samples	Myofibroblast	α-SMA^hi^, MYL9, MYLK, contractile	[87]
		Activated CAFs (2 subclusters; CAF1 and CAF2)	FAP, PDPN, PDGFRα; ECM-producing	
Lung cancer	Patient samples	Cluster 1	ECM-producing, TGF-β signature	[80]
		Cluster 2	α-SMA^hi^	
		Cluster 4	Enriched at leading edge	
		Cluster 5	High mTOR; enriched at tumor core	
		Cluster 7	High mTOR; enriched at leading edge	
Melanoma	Murine tumors (B16-F10)	S1–immune CAFs	CD34^hi^, CXCL12, C3, immunosuppressive	[90]
		S2–desmoplastic CAFs	CD34^lo^, CTGF, TNC; PDGFRα, ECM-producing	
		S3–contractile CAFs	α-SMA^hi^, RGS5	
Breast cancer and ovarian cancer	Patient samples	CAF-S1	FAP^hi^, α-SMA^hi^, CXCL12, IL-6	[45,84,85,91]
		CAF-S2	Low/no marker expression; contractile	
		CAF-S3	α-SMA^lo^, FSP1, PDGFRβ+	
		CAF-S4	CD29^hi^, α-SMA^hi^, FAP^lo^	
Breast cancer	Patient samples	iCAF	CXCL12	[92]
		myCAF	α-SMA, FAP, PDPN, COL1A1, COL1A2	
Breast cancer	Murine tumors (MMTV-PyVT)	Vascular CAF (vCAF)	α-SMA, PDGFRβ; angiogenesis	[93,94]
		Matrix CAF (mCAF)	α-SMA^lo^, PDGFRα; ECM-producing	
		Cycling CAF (cCAF)	PDGFRβ^hi^, angiogenesis	
		Developmental CAF (dCAF)	PDGFRβ-, SCRG1, SOX9; differentiation	
Breast cancer	Murine tumors (4T1)	PDPN-CAF	6 subclusters	[95]
		S100A4-CAF	2 subclusters	
Bladder cancer	Patient samples	Myo-CAF	RGS5, MYL9, MYH11	[83]
		iCAF	PDGFRα, CXCL12, IL-6, CXCL14, CXCL1, CXCL2	
Prostate cancer	Patient samples	CAF-S1	α-SMA, PDGFRβ	[86]
		CAF-S2	PDGFRα, PLAGL1	
		CAF-S3	α-SMA, HOXB2, MAFB	
Cholangiocarcinoma	Patient samples, Murine tumors (KRAS^G12D^/p19-induced, YAP^S127A^/AKT-induced)	myCAF	COL1A1, α-SMA	[82]
		iCAF	COL8A1, COL15A1, SERPINF1	
		mesCAF	CXCL12, HGF, RGS5 Mesothelin	

lo: low level, hi: high level, TAGLN: transgelin, MHCII: major histocompatibility complex class II, DCN: decorin, MYL: myosin light chain, CTGF: connective tissue growth factor, TNC: tenascin-C, RGS5: regulator of G protein signaling 5, FSP1: fibroblast-specific protein-1, SCRG1: stimulator of chondrogenesis 1, SOX9: SRY-box transcription factor 9, PLAGL1: pleomorphic adenoma gene 1, HOXB2: homeobox B2, MAFB: musculoaponeurotic fibrosarcoma oncogene homolog B, SERPINF1: serpin family F member 1.
